# Supplementary material for: A Point-of-Care Faecal Test Combining Four Biomarkers Allows Avoidance of Normal Colonoscopies and Prioritizes Symptomatic Patients with a High Risk of Colorectal Cancer
Source: Cancers (Basel). 2023 Jan 24;15(3):721. doi: 10.3390/cancers15030721 (PMC9913693; doi:10.3390/cancers15030721)
Supplement: Supplementary file 1 [file cancers-15-00721-s001.zip › cancers-2154379-supplementary.pdf]

Table S1. Median values of FIT and FC results in each diagnosis considered in the study.

	FIT<br>Median (IQR) µg/g	p value	FC<br>Median (IQR) µg/g	p value
Significant Pathology	17.19 (90.13)	**< 0.01**	82.23 (106.73)	**< 0.001**
No Significant Pathology	0.5 (2.90)		43.79 (90.87)	
Colorectal Cancer	**67.87** (539.83)	0.278 CRC vs IBD<br>**0.01 CRC vs ARS[1]**<br>**< 0.01 CRC vs ANRS[2]**<br>**< 0.01 CRC vs Normal**	121.13 (114.19)	0.126 CRC vs IBD<br>**0,04 CRC vs ARS[1]**<br>**0,01 CRC vs ANRS[2]**<br>**< 0.001 CRC vs Normal**
Inflammatory Bowel Disease	30.4 (120.3)	**0.024 IBD vs ARS[1]**<br>**< 0.01 IBD vs ANRS[2]**<br>**< 0.01 IBD vs Normal**	**149.95** (390.31)	**0,001 IBD vs ARS[1]**<br>**0,005 IBD vs ANRS[2]**<br>**< 0.001 IBD vs Normal**
Adenoma Requiring Surveillance	9.6 (42.59)	**< 0.01 ARS[1] vs ANRS[2]**<br>**< 0.01 ARS[1] vs Normal**	64.33 (111.87)	0.309 ARS[1] vs ANRS[2]<br>**0.043 ARS[1] vs Normal**
Adenoma Not Requiring Surveillance	0 (1.9)	0,106 ANRS[2] vs Normal	49.55 (90.69)	0.268 ANRS[2] vs Normal
Normal Colonoscopies	0.5 (2.90)		43.25 (85.93)	

[1] ARS: adenoma requiring surveillance.

[2] ANRS: adenoma not requiring surveillance.

Table S2. Diagnostic accuracy of FIT, FC, and the extreme results of the combined POC test for diagnosis of significant pathology stratified by presenting symptom.

Symptom	Test	True Positives	False Negatives	Sensitivity	Specificity	PPV	NPV
Rectal bleeding (n = 36/165)	FIT	25	11	69.4%	88.4%	62.5%%	91.2%
	FC	24	12	66.7%	61.2%	32.4%	86.8%
	≥1 test	33	3	91.7%	44.5%	31.7%	95.1%
	4 tests	11	25	30.6%	97.7%	78.6%	83.4%
Chronic diarrhea (n = 18/114)	FIT	7	11	38.9%	90.6%	43.7%	88.8%
	FC	9	9	50%	52.1%	16.4%	84.8%
	≥1 test	13	5	72.2%	46.9%	20.3%	90%
	4 tests	4	14	22.2%	100%	100%	87.3%
Abdominal pain (n = 17/97)	FIT	10	7	58.8%	96.2%	76.9%	91.7%
	FC	14	3	82.3%	56.2%	28.6%	94.7%
	≥1 test	16	1	94.1%	57.5%	32%	97.9%
	4 tests	4	13	23.5%	100%	100%	86%
Change in bowel habits (n = 16/91)	FIT	9	7	56.2%	82.7%	40.9%	89.9%
	FC	13	3	81.2%	57.3%	28.9%	93.5%
	≥1 test	15	1	93.8%	36%	23.8%	96.4%
	4 tests	3	13	18.8%	98.7%	75%	85.1%
Anemia / Iron deficiency (n=29/89)	FIT	16	13	55.2%	81.7%	59.3%	79%
	FC	22	7	75.9%	35%	36.1%	75%
	≥1 test	28	1	96.5%	26.7%	38.9%	94.1%
	4 tests	6	23	20.7%	96.7%	75%	71.6%

Table S3. Diagnostic accuracy of FIT, FC, and the extreme results of the combined POC test for diagnosis of CRC stratified by presenting symptom.

Symptom	Test	True Positives	False Negatives	Sensitivity	Specificity	PPV	NPV
Rectal bleeding (n = 7/165)	FIT	7	0	100%	79.1%	17.5%	100%
	FC	6	1	85.7%	56.9%	8.1%	98.9%
	≥1 test	7	0	100%	38.6%	6.7%	100%
	4 tests	6	1	85.7%	94.9%	42.8%	99.3%
Chronic diarrhea (n = 3/114)	FIT	3	0	100%	88.3%	18.8%	100%
	FC	2	1	66.7%	52.3%	3.7%	98.3%
	≥1 test	3	0	100%	45.1%	4.7%	100%
	4 tests	1	2	33.3%	97.3%	25%	98.2%
Abdominal pain (n = 6/97)	FIT	5	1	83.3%	91.2%	38.5%	98.8%
	FC	5	1	83.3%	51.7%	10.2%	97.9%
	≥1 test	6	0	100%	51.7%	12%	100%
	4 tests	2	4	33.3%	97.8%	50%	95.7%
Change in bowel habits (n = 5/91)	FIT	3	2	60%	77.9%	13.6%	97.1%
	FC	4	1	80%	52.3%	8.9%	97.8%
	≥1 test	5	0	100%	32.6%	7.9%	100%
	4 tests	2	3	40%	97.7%	50%	96.6%
Anemia / Iron deficiency (n=8/89)	FIT	5	3	62.5%	72.8%	18.5%	95.2%
	FC	7	1	87.5%	33.3%	11.5%	96.4%
	≥1 test	8	0	100%	21%	11.1%	100%
	4 tests	4	4	50%	95.1%	50%	95.1%

Table S4. AUCs (95%CI) of FIT, FC and each biomarker of the combined POC faecal test and its combination for the diagnosis of CRC, IBD and adenoma requiring surveillance.

	**Colorectal Cancer**	**Inflammatory Bowel Disease**	**Adenoma Requiring Surveillance**
FIT	0.838 (0.742-0.934)	0.859 (0.798-0.920)	**0.709 (0.637-0.780)**
FC	0.725 (0.635-0.816)	0.803 (0.678-0.928)	0.545 (0.466-0.624)
FIT or FC	0.775 (0.694-0.856)	0.807 (0.690-0.925)	0.581 (0.507-0.654)
hHb	0.832 (0.758-0.905)	0.857 (0.778-0.936)	0.691 (0.610-0.772)
hTf	0.707 (0.601-0.815)	0.735 (0.595-0.876)	0.593 (0.508-0.679)
hCp	0.685 (0.607-0.762)	0.714 (0.626-0.802)	0.574 (0.496-0.652)
hLf	0.746 (0.639-0.853)	0.911 (0.834-0.986)	0.489 (0.408-0.507)
Combination of 4 tests	**0.872 (0.815-0.929)**	**0.942 (0.911-0.974)**	0.673 (0.599-0.747)
